# Sperm quality parameters, fertilizing potential, metabolites, and DNA methylation in cold-stored and cryopreserved milt from Atlantic salmon (*Salmo salar* L.)

**DOI:** 10.3389/fgene.2023.1199681

**Published:** 2023-08-24

**Authors:** Birgitte Narud, Abdolrahman Khezri, Teklu T. Zeremichael, Anne-Lene Eriksen, Inger S. Grevle, Anna Nordborg, Geir Klinkenberg, Robert C. Wilson, Elisabeth Kommisrud

**Affiliations:** ^1^ Department of Biotechnology, Inland Norway University of Applied Sciences, Hamar, Norway; ^2^ Cryogenetics AS, Hamar, Norway; ^3^ SINTEF, Trondheim, Norway

**Keywords:** Atlantic salmon, spermatozoa, cold storage, cryopreservation, metabolite, RRBS

## Abstract

Cold storage and freezing/thawing of milt may affect sperm functionality and the subsequent fertilization ability of milt. This study aimed to investigate sperm quality parameters and fertilization potential of Atlantic salmon milt, stored cold and subsequently cryopreserved, using different storage conditions. The objective was also to assess if analysis of milt metabolites and sperm DNA methylation signatures could be applicable to further elucidate sperm quality and fertilization following preservation. Milt samples were collected from eight mature Atlantic salmon males and stored for 4 days at 2°C and 8°C. Samples were taken on day one of storage at 2°C and on day four of storage at 2°C and 8°C. Storage for 4 days at 8°C is expected to be detrimental to sperm quality, and was included to create contrasts. Correspondingly, aliquots of cold-stored milt were prepared for cryopreservation, resulting in a total of six experimental conditions. Samples from all six experimental conditions were used in fertilization trials and analyzed for sperm viability, motility, ATP content, DNA fragmentation index, and High DNA stainability. In addition, milt samples from four of the males were analyzed for targeted metabolites and DNA methylation signatures by reduced representation bisulfite sequencing. The fertilization trials were performed using sperm:egg ratios of 75 × 10^3^ and 500 × 10^3^, respectively. Storage duration, temperature, and cryopreservation of cold-stored milt influenced several sperm quality parameters, metabolites, and DNA methylation signatures. The total motility, progressive motility, ATP, and velocity parameters were the sperm parameters with the strongest correlation to fertilization rates (*p* < 0.01). Several metabolites were correlated with fertility rates in both cold-stored and cryopreserved samples (*p* < 0.05). The fertilizing capacity of cold-stored milt was significantly reduced after 4 days of storage at 8°C, while corresponding cryopreserved milt showed reduced fertilization at both storage temperatures (2°C and 8°C) (*p* < 0.05). The results indicate that cryopreservation of milt stored for 1 day does not compromise either fertilization ability or DNA methylation signatures.

## 1 Introduction

Artificial fertilization (AF) using cryopreserved milt has proven advantageous in aquaculture, as it facilitates cross-fertilizing fish spawning in different geographical locations or at different times (Blaxter, 1953). Furthermore, cryopreservation allows for the improvement of broodstock management at hatcheries and preservation of genetically selected breeds as well as endangered aquatic species (Asturiano et al., 2017). Thus, cryopreservation of fish gametes could be a valuable method for increased breeding progress. The large diversity of fish species, differences in reproductive physiology, and a relatively low focus on fish breeding may have limited the research interest in cryopreservation of milt (Tiersch et al., 2007; Cabrita et al., 2010). Nevertheless, aquaculture is a rapidly developing industry, with a growing focus on cryobanking services for several salmonids and other fish species (Asturiano et al., 2017).

In salmonids, cold storage of milt at 2°C–4°C is commonly used and an alternative to cryopreservation (Trigo et al., 2015; Merino et al., 2017). However, this method limits flexibility compared to long-term storage in liquid nitrogen. It will be beneficial for the aquaculture industry if cold storage of milt can be followed by cryopreservation, allowing for increased flexibility and applicability of cryopreservation (Kommisrud et al., 2020). Correct handling and storage of sperm in fish production are important, as several factors can affect sperm quality and hence the paternal contribution to the offspring (Herráez et al., 2017).

It is well known that both cold storage and freezing/thawing of spermatozoa may affect factors such as sperm plasma membrane integrity, motility, ATP content, and DNA integrity (Cabrita et al., 2005; Balamurugan et al., 2019; Kommisrud et al., 2020). Furthermore, these parameters are recognized as important factors for sperm cells to reach and fertilize the eggs and for proper embryo development (Liu et al., 2007; Cabrita et al., 2008; Pérez-Cerezales et al., 2010; Dzyuba et al., 2017). Thus, *in vitro* sperm quality assessment is crucial to objectively compare different preservation protocols and to select the samples most suitable for freezing and fertilization. However, different external factors (e.g., temperature, nutrition, toxins) may affect fertility by inducing molecular alterations undetectable by traditional *in vitro* assays (Riesco and Robles, 2013; Herráez et al., 2017). To broaden current knowledge regarding factors affecting reproductive success in fish, different omics techniques have been introduced (Egea et al., 2014), such as epigenomics and metabolomics (Labbé et al., 2017; Dietrich et al., 2019a).

Metabolites are defined as intermediates or final products of metabolic pathways and play significant roles in sperm physiology (Dietrich et al., 2019a). In mammals, several studies have been conducted to investigate the relationship between metabolites in semen and fertility (Zhao et al., 2018; Engel et al., 2019; Menezes et al., 2019; Narud et al., 2020). Though there are studies in fish investigating the metabolome of different cells, tissues, and biofluids [reviewed by Samuelsson and Larsson (2008); Dietrich et al. (2019a)], few have investigated metabolites in fish milt (Dietrich et al., 2019b; Tsentalovich et al., 2020).

The mammalian sperm epigenome is quite well described and reviewed previously by Champroux et al. (2018), and recently, there has been an increase in epigenetic studies performed in fish gametes and embryos (Labbé et al., 2017; Woods et al., 2018; Depincé et al., 2020). Environmental factors such as temperature and water quality, and reproductive biotechnologies including cryopreservation and AF, are examples of factors that may cause epigenetic modifications in fish gametes and embryos (Labbé et al., 2017).

The main objective of this study was to assess if cold storage of milt can be followed by cryopreservation without compromising post-thaw sperm quality and the fertilizing potential in Atlantic salmon (*Salmo salar* L.). Another goal was to investigate if analysis of milt metabolites and sperm DNA methylation signatures could be applicable to further elucidate sperm quality and fertilization following preservation. To obtain the aims, milt was stored at two different temperatures, 2°C and 8°C, where 8°C was hypothesized to be detrimental to sperm quality. Furthermore, two different sperm:egg ratios were employed in the fertilization trials.

## 2 Materials and methods

### 2.1 Sample collection and preservation

Milt from eight mature Atlantic salmon males kept at a broodstock facility in Central Norway was collected by gonad extraction, during spawning season. Briefly, males were culled, and the gonads were removed, cleaned, and homogenised using a mechanical grinder. The homogenate was weighed and a 1:1 amount of AquaBoost^®^ SpermCoat (Cryogenetics AS, Hamar, Norway) was added, before filtration and storage in 650 mL cell culture flasks (VWR International, Radnor, PA, United States). The milt samples were shipped overnight to the laboratory (Cryogenetics AS, Hamar, Norway) while kept cold on paper on top of crushed ice to ensure a temperature of about 2°C on arrival. Aliquotes of milt from each male (10 mL) were stored at 2°C and 8°C in cell culture flasks (50 mL) with filter screw caps (VWR International, Radnor, PA, United States) for 4 days. Samples were taken on day one of storage at 2°C (hereafter referred to as D1-2°C) and on day four of storage at 2°C and 8°C (hereafter referred to as D4-2°C, D4-8°C). Correspondingly aliquots of cold-stored milt were prepared for cryopreservation (hereafter referred to as D1-2°C/Cryo, D4-2°C/Cryo, D4-8°C/Cryo), resulting in a total of six experimental conditions. Samples from all six experimental conditions were analyzed for sperm viability, motility, ATP content, DNA fragmentation index (DFI), and High DNA stainability (HDS). In addition, samples from four of the males, selected based on their contrasting performance in the fertilization trials (described in 2.7), were analyzed for sperm metabolites and DNA methylation signatures by reduced representation bisulfite sequencing (RRBS). Cryopreservation was conducted using the following procedure. Sperm concentration was estimated after mixing 10 µL milt with 4 mL 0.9% NaCl using an SDM6 photometer (Minitüb GmbH, Tiefenbach, Germany) at 546 nm using a pre-determined calibration curve for Atlantic salmon sperm. Sperm concentration varied between 10.2 and 12.2 × 10^9^ sperm/mL. Based on the concentration estimate, the milt was diluted using a proprietary fish sperm extender (Cryogenetics AS, Hamar, Norway) to a standardized sperm concentration. Pre-printed 0.5 mL medium straws (IMV technologies, L’Aigle, France) were filled with milt and sealed with ultrasound using an MPP Uno automated filling and sealing machine (Minitüb GmbH, Tiefenbach, Germany). Cryopreservation was performed according to a proprietary protocol (Cryogenetics AS, Hamar, Norway) before transfer and storage in liquid N_2_. The preprint ensured identification of the male, storage condition, and date of cryopreservation.

### 2.2 Experimental setup *in vitro* analyses

Samples from the six different experimental conditions from all individuals were treated identically according to the following procedure. Samples were analyzed in triplicate for viability, ATP content, and DFI, and in duplicate for motility assessment. For the cryopreserved milt, three doses per batch were thawed in a water bath (25°C, 30 s) and pooled. All samples were kept cold on paper on top of crushed ice before *in vitro* analysis. All chemicals were purchased from Sigma-Aldrich (St. Louis, MO, United States) unless otherwise noted.

### 2.3 Sperm plasma membrane integrity

The analysis of sperm plasma membrane integrity (sperm viability) was assessed using propidium iodide (PI, L-7011, LIVE/DEAD®Sperm Viability Kit, Molecular Probes, ThermoFisher Scientific, Waltham, MA United States). The spermatozoa were stained in dark (4°C, 10 min) in PBS (137 mM NaCl, 2.7 mM KCl, 1.76 mM KH_2_PO_4_, 8.1 mM Na_2_HPO_4_·2H_2_O, pH 7.4) staining solution with a final concentration of 1.5 × 10^6^ spermatozoa/mL and 0.48 mM PI. The flow cytometry analysis was performed using a Cell Lab Quanta ™ SC MPL flow cytometer (Beckman Coulter, Fullerton, CA, United States), equipped with a 22 mW argon laser with an excitation wavelength of 488 nm. Data were analyzed using Cell Lab Quanta SC MPL Analysis software program (Software Version 1.0, Beckman Coulter, Fullerton, CA, United States). Side scatter (SS) signals were used for the identification of spermatozoa, and PI fluorescence in SS-gated spermatozoa was detected using a 670 nm long pass (LP) filter (FL3).

### 2.4 Sperm motility analysis by CASA

Sperm motility analysis was performed using a SCA evolution CASA system (Sperm Class Analyzer^®^ version 6.1, Microptic SL, Barcelona, Spain). The CASA system was equipped with a phase contrast Eclipse Ci-S/Ci-L microscope (Nikon, Japan) with a temperature-controlled stage using a water circulation pump, and a Basler digital camera (Basler Vision Technologies, Ahrensburg, Germany). Chamber slides were kept on the cooled microscopic stage before analysis. Samples were diluted to a working sperm concentration of 87 × 10^6^ cells/mL in AquaBoost^®^ Dilutor (Cryogenetics AS, Hamar, Norway) prior to analysis. Sperm activation was initiated by the addition of AquaBoost^®^ Activator (Cryogenetics AS, Hamar, Norway) simultaneously with loading the sample to the microscope slide. Using a Picus (0.2–10 µL) electronic 1-channel pipette (Sartorius Stedim Biotech GmbH, Göttingen, Germany) in Multi-Aspirating Mode, aspiration was done sequentially in the following order: 1.2 µL diluted milt—air—1.2 µL AquaBoost^®^ Activator. For each CASA measurement, the aspiration volume was dispensed into a single chamber on a Leja 20 µm 4 chamber microscope slide (Leja, Nieuw-Vennep, the Netherlands). Image analysis was performed on four consecutive fields within approximately 8 s after activation, with a total of at least 400 cells analyzed per sample. The parameters recorded were total motility (TM, %), progressive motility (PM, %), straight-line velocity (VSL, mm/sec), average path velocity (VAP, mm/sec), curvilinear velocity (VCL, mm/sec), straightness (STR, ratio VSL/VAP) and linearity (LIN, ratio VSL/VCL). Sperm cells were identified by sperm head area of 10–50 μm^2^, a 50 Hz frame rate was used, and 45 frames were captured per object. Motile sperm cells were defined based on VAP (µm/sec) 15< Slow < 30 < Medium < 50 < Rapid. Cells were classified as progressive motile if STR >45%.

### 2.5 Sperm ATP content

The sperm ATP content was determined using the CellTiter-Glo^®^ Luminescent Cell Viability Assay (G7571, Promega, Madison, WI, United States), a FLUOstar OPTIMA multiwell plate reader (BMG Labtech GmbH, Offenburg, Germany) and MARS data analysis software (Version 1.10, BMG Labtech GmbH, Offenburg, Germany). In brief, milt samples were diluted to 5 × 10^6^ spermatozoa/mL in AquaBoost^®^ SpermCoat (Cryogenetics AS, Hamar, Norway), and each sample (50 µL) was transferred to wells in an opaque 96-well microtiter plate (NUNC™, Roskilde, Denmark). CellTiter-Glo^®^ Reagent (50 µL) was added to each well and the mixture was gently shaken for 2 min in a rotary shaker (300 rpm) (IKA^®^ MS 3 digital, IKA®-Werke GmbH & Co., Staufen, Germany), followed by 10 min incubation in room temperature (RT). The data recorded was measured in relative luminescence units (RLU), and converted to corresponding ATP values (nM) according to a prepared standard curve.

### 2.6 Sperm chromatin integrity

Sperm chromatin integrity was analyzed using the Sperm Chromatin Structure Assay (SCSA), as previously described for bovine semen (Narud et al., 2020), with minor modifications. In brief, milt samples were diluted to about 1.5 × 10^7^ spermatozoa/mL in TNE buffer (10 mM Tris-HCL, 0.1 M NaCl, 1 mM EDTA, pH 7.4) in a final volume of 200 μL. Next, 400 μL acid detergent solution [0.38 M NaCl, 120 mM HCL, 0.1% (w/v) Triton X-100, pH 1.2] was added, followed by incubation for 30 s in RT. Next, 1.2 mL acridine orange (AO) staining solution [6 mg/mL AO (A3568, Life Technologies, OR, United States)] in a buffer containing 37 mM citric acid, 0.126 M Na_2_HPO_4_, 1.1 mM EDTA, and 0.15 M NaCl (pH 6) was added. Data acquisition started after a 3 min setup mode, in which 5000 events were captured for each sample at a rate of ∼200 events/sec. Analysis in FCS Express 6 Flow cytometry Software (Denovo Software, Los Angeles, CA, United States) revealed the percentage of red (ssDNA) and green (dsDNA) fluorescence. Based on a histogram of the fluorescence ratio red/(red + green), the sperm cells with fragmented DNA (DFI, %) were calculated. The percentage of spermatozoa with high DNA stainability (HDS, %) was determined by the bivariate cytogram.

### 2.7 Fertilization trials

Fertilization trials were performed using milt from all six different experimental conditions (2.1). For each trial, eggs from one female were stripped and shipped overnight on ice from a broodstock facility to Cryogenetics AS in Hamar. Eggs were kept in ovarian fluid and shipped in 650 mL cell culture flasks (filled with approximately 300 mL per flask). *In vitro* fertilization was carried out using two different sperm:egg ratios, 75 × 10^3^ sperm cells/egg (duplicates) and 500 × 10^3^ sperm cells/egg. Each fertilization unit consisted of approximately 150 eggs estimated by weight (18 g). Cryopreserved straws were thawed for 30 s in a water bath at 25°C. Fertilization was performed in a climate-controlled room at 4°C. The temperature difference between eggs and other media throughout the fertilization trial was at all times below 2.5°C. For each fertilization, sperm and eggs were gently mixed in a plastic cup while adding AquaBoost^®^ Activator sperm activating solution to slightly cover the eggs. After 2 min, eggs were rinsed in physiological saline solution (0.9% NaCl) and transferred to nylon mesh-bottomed incubation rings. Incubation rings were then incubated in fresh water. After incubation for approximately 150–200 h-degrees post-fertilization, eggs were transferred to a plastic cup and AquaBoost^®^ Quattro (Cryogenetics AS, Hamar, Norway) was added. After fixation for 15 min in this solution, each egg was visually inspected and categorized as fertilized or unfertilized, based on the presence or absence of cell division.

### 2.8 Analysis of targeted metabolites in fish milt

Samples from all six experimental conditions were analyzed for targeted metabolites. For the cold-stored samples, 2 mL milt prediluted 1:1 with AquaBoost^®^ SpermCoat was centrifuged (800 × g, 10 min) to separate the supernatant (seminal plasma and extender, hereafter referred to as seminal plasma) from the pellet (sperm cells). For the cryopreserved samples, one straw of frozen-thawed milt was centrifuged (500 × g, 5 min). All pellet and supernatant samples were snap-frozen in liquid N_2_ and shipped on dry ice to SINTEF (SINTEF Industry, Trondheim, Norway) for analyses of targeted amines and amino acids. Samples of the proprietary extenders used were also analyzed for background levels of the targeted metabolites.

At SINTEF the samples were thawed, and an aliquot was collected for sample preparation and analysis. Sample preparation consisted of protein precipitation by the addition of four volumes of ice-cold methanol. Following centrifugation, a 100 μL aliquot of the supernatant was collected for amine analysis, dried using a speed-vac, and dissolved in an aqueous internal standard mix prior to analysis. A 10 μL aliquot of the supernatant was collected for amino acid analysis. The sample was mixed with the amino acid internal standard mixture and derivatized using an AccQ-Tag Derivatization Kit (Waters, Milford, MA, United States) prior to analysis.

Metabolite analysis of amino acids and amines was performed on an Agilent 1290 Infinity II LC system (Agilent, Santa Clara, United States) coupled to an Agilent 6495 QqQ mass spectrometer, using one method for amino acids and one for amines. The QqQ-MS was equipped with a jet-stream ESI source operated in positive mode. The QqQ-MS was operated in dynamic multiple-reaction monitoring (MRM) mode (delta Rt = 1 min) with a unit mass resolution for both mass filters. The MRM transitions for standards and internal standards and the employed collision energies, gas temperatures, and flows are given in Supplementary Tables S1, S2. For the amines (choline, creatine, and L-carnitine), the chromatographic separation was performed in HILIC mode employing a BEH Amide (2.1 × 150 mm, 2.7 μM) column (Waters, Milford, United States) and gradient elution using an 80:20 mixture of 25 mM Formic acid and 50 mM ammonium acetate as eluent A, and acetonitrile as eluent B, at a flow rate of 0.3 mL/min. The gradient used started at 90% B and was decreased stepwise to 5% B at 6.5 min. The column thermostat was maintained at 35°C and the autosampler at 8°C. The injection volume was 1 μL. Mixed standards at 0, 0.5, 1, 10, 50, 100, 500, 1,000, and 5,000 nM were used for calibration and quantitation. Amino acids (alanine, arginine, asparagine, GABA, glutamate, glutamine, glycine, Histidine, isoleucine, leucine, lysine, methionine, ornithine, phenylalanine, proline, serine, threonine, tryptophan, tyrosine, and valine) were analyzed in reversed-phase mode employing an Ascentis Express C18 (2.1 × 150 mm, 2.7 μM) column (Sigma-Aldrich), and gradient elution using 25 mM Formic acid as eluent A, and acetonitrile as eluent B, at a flow rate of 0.3 mL/min. The gradient used started at 5% B and was increased stepwise to 50% B at 15 min. The column thermostat was maintained at 20°C and the autosampler at 6°C. The injection volume was 2 μL. Mixed standards at 0, 0.1, 1, 5, 10, 25, 50, 100, 250, 500, and 1,000 μM were used for calibration and quantitation.

### 2.9 RRBS library preparation and illumina sequencing

Parallel samples from four of the eight individuals belonging to D1-2°C, D1-2°C-Cryo, D4-2°C, and D4-8°C groups were considered for the construction of RRBS libraries using a gel-free multiplexed technique (Boyle et al., 2012), previously optimized to study sperm DNA methylation in boar (Khezri et al., 2019) and bull (Khezri et al., 2020). In brief, 100 ng genomic DNA isolated from milt samples was digested overnight at 37°C using *MspI* and *Taqα1* enzymes (New England Biolabs). Fragmented DNA was subjected to Gap filling, A-tailing, and size selection (300–500 bp). Size selected DNA samples were adapter-ligated using NEXTflex™ Bisulfite-Seq barcodes (Bio Scientific Corporation) and were bisulfite converted using EpiTect kit (QIAGEN, Germany) following the manufacturer’s protocol. The product was cleaned up according to recommendations in the QIAGEN EpiTect kit (Gu et al., 2011) and PCR amplified. The PCR product was further enriched by adding 1× SPRI AMPure XP beads. The DNA concentration of eluted and cleaned RRBS libraries was measured using the PicoGreen dsDNA absorbance method. The prepared libraries were sent to the Norwegian Sequencing Centre where sequencing was performed by Illumina HiSeq 4000 in paired-end (2 × 150 bp) mode.

### 2.10 Bioinformatic analyses

#### 2.10.1 Quality check and pre-processing of illumina reads

The quality of paired-end Illumina reads was checked using fastQC (v 0.11.8 for Linux). Illumina adapters and low-quality sequences (below 20 bp and Phred score of 30) were trimmed using the Trim-galore (v 0.4.4 for Linux) (Martin, 2011).

#### 2.10.2 Aligning the clean reads against Atlantic salmon reference genome

The Atlantic salmon reference genome (ICSASG_v2) was downloaded from the UCSC database and indexed using bismark_genome_preperation script in Bismark (v 0.19.0 for Linux) (Krueger and Andrews, 2011). Clean reads after trimming were collected and mapped against the indexed genome using bowtie2 aligner, integrated with Bismark using default parameters. Global CpG methylation level in Bismark was internally calculated for all covered cytosines (Cs) using the following formula: % of global methylation = 100 × number of methylated Cs/number of methylated Cs + number of unmethylated Cs.

#### 2.10.3 Differential methylation analysis

Differential methylated analysis was performed using the methylKit package (v 1.6.1) (Akalin et al., 2012) in Rstudio (v 1.1.453 for Linux). The analysis was performed for five different experiments (Experiments A to E). For each experiment, test and control groups were defined as described in Table 1.

**TABLE 1:** An overview of the different experiments (Exp A to E) conducted for differential methylation analysis. For each experiment, milt samples from four different salmon were considered. The same milt samples (treated differently) were considered for both the control and test.

	Control	Test
Exp A	D1-2°C	D1-2°C/Cryo
Exp B	D4-2°C	D4-8°C
Exp C	D1-2°C	D4-2°C
Exp D	D1-2°C/Cryo	D4-2°C
Exp E	D1-2°C/Cryo	D4-8°C

D1-2°C = cold-stored for 1 day at 2°C, D4-2°C = cold-stored for 4 days at 2°C, D4-8°C = cold-stored for 4 days at 8°C, D1-2°C/Cryo = cold-stored for 1 day at 2°C before cryopreservation.

For differential methylated analysis, only CpGs ≥ 10 × coverage depth (CpG_10x_) were considered, and reads containing CpGs with more than 99. Ninth percentile coverage were excluded. After applying logistic regression analysis with a sliding linear model to correct for multiple comparisons, only DMCs with ≥10% methylation difference and *q* value < 0.05 (filtered DMCs onwards) were considered for downstream analysis. In this study, hypermethylated and hypomethylated Cs are defined as differential methylation over 10% or smaller than −10% in the test group compared to the control group, respectively.

#### 2.10.4 Annotation of filtered DMCs with gene elements and CpG features

BED12 files containing gene and CpG annotation for the Atlantic salmon assembly (ICSASG_v2) were downloaded from the UCSC table browser (UCSC, 2018). The genomation package (v 1.14.0) in Rstudio was employed to annotate filtered DMCs (contained coordinate) with the nearest transcriptional start site (TSS), gene elements (exons, introns, promoters, and intergenic regions), and CpG features (CpG islands, CpG shores, other). In this study, promoters and CpG shores were defined as ±1,000 bp and ±2,000 bp of the TSS and CpG islands, respectively.

#### 2.10.5 Gene ontology and functional analyses of annotated genes

Corresponding transcript stable ID(s) to annotated TSSs were converted to UniProt accession ID using the gene ID conversion tool available from g: Profiler (Raudvere et al., 2019). Obtained UniProt accession IDs were submitted as a list to DAVID Bioinformatics resources for gene ontology functional annotation analysis (Huang da et al., 2009). The list was recognized automatically and DAVID assigned *Salmo salar* as the target organism. Furthermore, in order to get a better annotation and reduce database bios, multiple databases were selected for enrichment analyses: Interpro, KEGG pathway, Go terms including biological process, cellular components, molecular functions, SMART, and UniprotKB Keywords including biological process, cellular components, molecular functions. Gene enrichment for each identified term or pathway was calculated using Fisher’s exact test and the *p*-value was Benjamini corrected for multiple testing and set to 0.05.

### 2.11 Statistical analyses

The statistical analyses of the different *in vitro* sperm quality traits, metabolites, and fertility performance were performed using SAS Version 9.4 for Microsoft Windows (SAS Institute, Cary, NC, United States). The data were tested for normal distribution by the Shapiro-Wilk test. Parameters that did not show a normal distribution were log-transformed to approach a normal distribution prior to further statistical analysis. The mixed procedure in SAS was used to perform the least square means analyses. For the sperm quality parameters, the effect of the explanatory variables on the outcome variables; motility parameters, viability, DFI, HDS, and ATP, were estimated by the following mixed linear model: Y_ijk_ = µ + C_i_ + S_j_ + M_k_ e_ijk_, where: Y_ijk_ = observation of *in vitro* sperm parameter per semen sample; µ = overall mean of the *in vitro* sperm parameter; C_i_ = fixed effect of storage condition, i = 1 (day one milt), 2 (stored at two degrees) or 3 (stored at eight degrees); S_j_ = fixed effect of semen state, j = 0 (fresh) or 1 (frozen-thawed); M_k_ = random effect of male, k = 1 to 8; e_ijk_ = random error. The same model was used to perform a least square means analysis for the metabolites in sperm and seminal plasma, using the different amines and amino acids as outcome variables. For the fertilization outcome, the same model was used, however, the outcome variables were the fertilization outcome either from a sperm:egg ratio of 75 or 500 × 10^3^, respectively. The Tukey test was applied for pairwise comparisons between means. The correlation between *in vitro* sperm quality parameters or metabolites and fertilization outcome was assessed, where correlation coefficients (Spearman) were calculated. The significance level in all statistical tests was set to 0.05.

## 3 Results

### 3.1 Sperm viability

The sperm viability of cold-stored milt in D1-2°C was on average 95.1% ± 0.7 and decreased significantly in D4-2°C (*p* < 0.0001) and D4-8°C (*p* < 0.0002) (Table 2).

**TABLE 2:** *In vitro* sperm parameters of milt collected from eight Atlantic salmon (*Salmo salar* L.) males are presented as mean ± SD for cold-stored and cryopreserved milt. Milt was stored cold for 1 day at 2°C (D1-2°C) and 4 days at 2°C (D4-2°C) and 8°C (D4-8°C), respectively, with subsequent cryopreservation (D1-2°C/Cryo, D4-2°C/Cryo and D4-8°C/Cryo). Different superscripts denote significant differences (*p* < 0.05) between storage conditions within cold-stored and cryopreserved samples based on a linear mixed model. Asterix denotes significant differences (*p* < 0.05) between cold-stored and cryopreserved samples.

	Cold-stored			Cryopreserved		
	D1-2°C	D4-2°C	D4-8°C	D1-2°C/Cryo	D4-2°C/Cryo	D4-8°C/Cryo
Viability (%)	95.1 ± 0.7^a^	75.5 ± 8.2^b^	61.5 ± 12.9^c^	59.0 ± 7.7^a,^*	40.6 ± 8.4^b,^*	29.2 ± 6.6^c,^*
TM (%)	35.8 ± 11.5^a^	6.6 ± 4.9^b^	1.5 ± 2.1^b^	32.7 ± 9.1^a^	8.5 ± 4.8 ^b^	3.6 ± 2.6^b^
PM (%)	32.4 ± 11.4^a^	4.7 ± 3.6^b^	0.7 ± 1.3 ^b^	29.7 ± 9.3^a^	7.2 ± 4.5^b^	2.5 ± 2.3^b^
VAP (µm/s)	86.1 ± 7.8^a^	56.9 ± 6.4^b^	27.4 ± 12.5^c^	97.5 ± 10.1^a,^*	72.3 ± 10.5^b,^*	53.7 ± 14.0^c,^*
VCL (µm/s)	100.6 ± 8.9^a^	71.1 ± 6.5^b^	41.3 ± 12.7^c^	107.7 ± 9.7^a^	83.6 ± 9.5^b,^*	65.5 ± 14.9^c,^*
VSL (µm/s)	59.9 ± 8.7^a^	42.2 ± 6.8^b^	15.3 ± 11.8^c^	85.3 ± 9.5^a,^*	61.1 ± 10.6^b,^*	40.6 ± 11.6^c,^*
STR (%)	69.1 ± 7.1^a^	71.1 ± 5.3^a^	45.7 ± 16.2 ^b^	85.3 ± 2.3^a,^*	79.2 ± 6.1^a,^*	69.8 ± 8.8^b,*^
LIN (%)	59.2 ± 6.3^a^	55.5 ± 6.0^a^	32.3 ± 14.2^b,^*	75.5 ± 3.1^a,^*	66.0 ± 9.4^b,^*	56.5 ± 7.2^c,^*
ATP (nM)	428.5 ± 41.8^a^	132.8 ± 43.3^b^	19.7 ± 37.7^c^	436.9 ± 45.7^a^	154.9 ± 70.2^b^	37.6 ± 25.0^c^
DFI (%)	0.8 ± 0.1^a^	1.7 ± 0.7^b^	3.3 ± 0.5^c^	2.3 ± 0.5^a,^*	3.5 ± 0.6^b,^*	4.3 ± 1.0^c,^*
HDS (%)	1.9 ± 0.3^a^	5.0 ± 2.0^b^	10.0 ± 1.6^c^	2.8 ± 0.7^a^	5.7 ± 1.1^b^	7.0 ± 1.3^c,^*

TM, total motility; PM, progressive motility; VAP = velocity average path; VCL, velocity curvilinear; VSL, velocity straight-line; STR, straightness; LIN, linearity, DFI = DNA, fragmentation index; HDS, High DNA, stainability.

Frozen-thawed milt samples showed significantly lower viability than the cold-stored samples (*p* < 0.0001), for all experimental conditions. There was a significant higher post-thaw sperm viability in D1-2°C/Cryo compared to D4-2°C/Cryo and D4-8°C/Cryo samples (*p* < 0.002).

### 3.2 Sperm motility parameters

Sperm motility of cold-stored milt samples measured as both total (TM) and progressive (PM) motility, were higher in D1-2°C compared to D4-2°C and D4-8°C (*p* < 0.0001) (Table 2). The TM and PM measured in D4-2°C were not significantly different from D4-8°C. The sperm velocity parameters VAP, VCL, and VSL were higher in D1-2°C compared to D4-2°C and D4-8°C (*p* < 0.0005). The parameters STR and LIN were not significantly different between D1-2°C and D4-2°C. However, both parameters decreased in D4-8°C (*p* < 0.0001).

Cryopreservation of milt samples after cold storage did not affect the average sperm motility measured as TM and PM (*p* > 0.05). The post-thaw TM and PM were higher in D1-2°C/Cryo compared to D4-2°C/Cryo and D4-8°C/Cryo (*p* < 0.0001). Post-thaw TM and PM did not differ between D4-2°C/Cryo and D4-8°C/Cryo (*p* > 0.05). Average VAP, VSL, and VCL in frozen-thawed samples increased (*p* < 0.02) compared to cold-stored samples, except for VCL in D1-2°C/Cryo (*p* > 0.05). The velocity parameters VAP, VCL, and VSL were higher in D1-2°C/Cryo compared to D4-2°C/Cryo and D4-8°C/Cryo (*p* < 0.001). Samples cryopreserved after cold storage had higher post-thaw STR and LIN compared to cold-stored samples (*p* ≤ 0.05). The parameter LIN was lower in D4-2°C/Cryo and D4-8°C/Cryo compared to D1-2°C/Cryo (*p* < 0.03). Post-thaw STR decreased in D4-8°C/Cryo compared to D1-2°C/Cryo and D4-2°C/Cryo (*p* < 0.0005).

### 3.3 Sperm ATP content

Sperm ATP content was significantly lower in D4-2°C compared to D1-2°C and was further reduced in D4-8°C (*p* < 0.0001) (Table 2). The ATP content of frozen-thawed milt samples was not significantly different from the cold-stored samples. Post-thaw ATP content was lower in D4-2°C/Cryo compared to D1-2°C/Cryo and was further reduced in D4-8°C/Cryo (*p* < 0.0001).

### 3.4 Sperm chromatin integrity by SCSA

Higher percentages of the sperm chromatin integrity parameters DFI (*p* < 0.003) and HDS (*p* < 0.0001) were observed in milt from D4-2°C and D4-8°C compared with D1-2°C. The percentages of DFI and HDS were higher in D4-8°C compared to D4-2°C (*p* < 0.0001).

Samples cryopreserved after cold storage showed a higher percentage of DFI compared to cold-stored samples (*p* < 0.05). The post-thaw percentages of DFI and HDS were higher in D4-2°C/Cryo compared to D1-2°C/Cryo (*p* < 0.0001) and was further increased in D4-8°C (*p* < 0.05).

### 3.5 Fertilization

**FIGURE 1:**
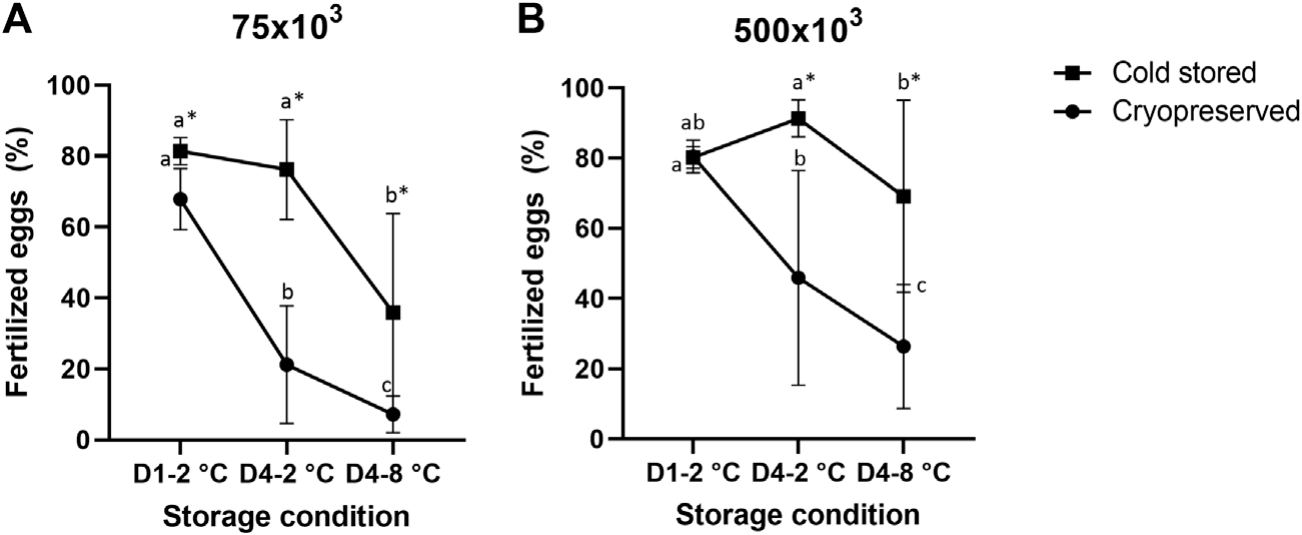
Percentage of fertilized eggs (mean ± SD after fertilization with cold stored or frozen-thawed sperm stored cold for 1 day at 2°C (D1-2°C) and 4 days at 2°C and 8°C (D4-2°C and D4-8°C) prior to cryopreservation. Fertilizations were carried out in triplicate with sperm:egg ratios of 75 × 10^3^ **(A)** or 500 × 10^3^ **(B)**. Different letters denote significant differences (*p* < 0.05) between storage conditions within cryopreserved or cold-stored samples, respectively. Asterix denotes significant differences (*p* < 0.05) between cold-stored and cryopreserved samples within the same storage condition.

There was a significant influence of cold storage duration and temperature using both sperm:egg ratios (Figure 1). For the samples cryopreserved after cold storage, there was a reduction in fertilized eggs from D1-2°C/Cryo to D4-2°C/Cryo and a further reduction in D4-8°C/Cryo (*p* < 0.05). The cold stored samples in D4-2°C were not significantly different from D1-2°C. However, D4-8°C samples significantly reduced the number of fertilized eggs. Overall, cryopreservation of cold-stored samples resulted in a significant reduction of fertilized eggs compared to cold-stored samples, except for D1-2°C using sperm:egg ratio of 500 × 10^3^.

### 3.6 Correlation of sperm parameters and fertilization rate

Spearman correlations between all *in vitro* sperm parameters and fertilization rate were significantly different from zero (*p* < 0.05) using a sperm:egg ratio of 75 × 10^3^ for both cold-stored milt and milt cryopreserved after cold storage (Supplementary Table S3). The highest correlations were found for the motility parameters TM (r_s_ = 0.96) and PM (r_s_ = 0.96), ATP (r_s_ = 0.93) content, and the kinematic parameters VAP (r_s_ = 0.91), VCL (r_s_ = 0.91) and VSL (r_s_ = 0.91) of frozen-thawed samples. The post-thaw chromatin integrity parameters DFI (r_s_ = −0.82) and HDS (r_s_ = −0.66) were negatively correlated with the fertilization outcome. No significant correlations were found for cold-stored samples using sperm:egg ratio of 500 × 10^3^. For the frozen-thawed samples, all correlations were weaker for fertilizations with a sperm:egg ratio of 500 × 10^3^ compared with 75 × 10^3^ (Supplementary Table S3).

### 3.7 Effect of storage on metabolite concentrations

The effects of storage duration and temperature on amine and amino acid concentrations were assessed in sperm cells (Table 3) and seminal plasma (Supplementary Table S4) from all experimental conditions. For the cold-stored sperm samples, the concentration of the amines choline and creatine increased in D4-2°C and D4-8°C compared with samples in D1-2°C (*p* < 0.05). The concentration of L-carnitine increased from D1-2°C to D4-2°C (*p* < 0.0001) but was not further increased in D4-8°C (*p* > 0.05). There was a significant overall effect of cryopreservation on all amine concentrations (*p* < 0.002). The concentration of choline was not significantly affected by storage duration and temperature in samples cryopreserved after cold storage. The concentration of carnitine/L-carnitine was significantly reduced from D1-2°C/Cryo to D4-2°C/Cryo but was not further reduced in D4-8 °C/Cryo.

**TABLE 3:** Mean ± SD for amine and amino acid concentrations (µM) in sperm cells of cold-stored milt and milt cryopreserved after cold storage. Samples were collected from four Atlantic salmon (*Salmo salar* L.) males. Milt was stored cold for 1 day at 2°C (D1-2°C) and 4 days at 2°C (D4-2°C) and 8°C (D4-8°C), respectively, with subsequent cryopreservation (D1-2°C/Cryo, D4-2°C/Cryo and D4-8°C/Cryo). Different superscripts denote significant differences (*p* < 0.05) between storage days and temperature within cold-stored and cryopreserved samples based on a linear mixed model.

	Cold-stored			Cryopreserved		
Amines	D1-2°C	D4-2°C	D4-8°C	D1-2°C/Cryo	D4-2°C/Cryo	D4-8°C/Cryo
Choline	90.5 ± 11.6^a^	115.5 ± 17.9^b^	142.5 ± 18.1^c^	17.8 ± 0.7	23.1 ± 0.9	24.3 ± 1.9
Creatine	9371.2 ± 921.4^a^	7132.2 ± 400.7^b^	5411.3 ± 795.9^c^	2195.3 ± 490.6^a^	845.2 ± 144.0^b^	718.9 ± 51.1^b^
L-Carnitine	79.0 ± 3.8^a^	139.0 ± 32.9^b^	139.7 ± 21.9^b^	40.4 ± 10.8^a^	15.7 ± 2.8^b^	13.7 ± 1.1^b^
Amino acids						
Alanine	319.8 ± 94.9^a^	509.0 ± 72.4 ^b^	541.6 ± 65.8 ^b^	211.7 ± 30.6^a^	121.8 ± 14.6 ^b^	93.1 ± 9.2 ^b^
Arginine	1140.5 ± 454.4^a^	4288.7 ± 687.2^b^	6855.6 ± 1562.8^c^	37.4 ± 2.7	297.2 ± 45.7	700.8 ± 154.0
Asparagine	42.2 ± 26.0^a^	24.3 ± 6.7^b^	7.4 ± 2.6^c^	3.8 ± 1.2	0.6 ± 0.3	0.1 ± 0.1
GABA	25.0 ± 5.9^a^	19.5 ± 3.9^b^	12.6 ± 1.9^c^	7.5 ± 1.4^a^	2.2 ± 0.7^b^	1.5 ± 0.2^b^
Glutamate	414.3 ± 12.1^a^	431.7 ± 21.5 ^ab^	464.2 ± 33.3^b^	87.8 ± 12.0	88.5 ± 15.8	99.1 ± 6.1
Glutamine	243.4 ± 71.1^a^	339.3 ± 56.8^b^	290.6 ± 48.4 ^ab^	46.2 ± 5.7	34.9 ± 4.0	24.9 ± 1.4
Glycine	4306.2 ± 632.4	4041.3 ± 523.6	3805.1 ± 664.3	956.2 ± 122.2	486.1 ± 73.1	497.2 ± 41.7
Histidine	98.9 ± 26.7^a^	130.9 ± 20.1^b^	122.4 ± 24.0 ^ab^	26.8 ± 3.9	20.7 ± 2.5	15.5 ± 1.6
Isoleucine	96.1 ± 49.9	94.9 ± 24.0	62.0 ± 25.6	41.1 ± 5.1	29.6 ± 6.0	20.8 ± 6.8
Leucine	330.5 ± 67.3	366.4 ± 61.4	375.1 ± 77.0	94.6 ± 15.1	60.9 ± 6.9	42.0 ± 5.9
Lysine	114.1 ± 23.5^a^	155.1 ± 19.6 ^b^	158.4 ± 27.2^b^	28.5 ± 1.3	20.5 ± 2.0	18.0 ± 2.7
Methionine	99.6 ± 23.0	116.0 ± 13.6	117.2 ± 17.0	31.0 ± 5.9	22.0 ± 1.6	14.7 ± 1.7
Ornithine	14.7 ± 3.9^a^	99.7 ± 34.4 ^b^	182.1 ± 56.5^c^	1.9 ± 0.6	15.5 ± 6.4	27.4 ± 9.5
Phenylalanine	115.0 ± 21.5^a^	142.4 ± 19.0^b^	142.4 ± 23.3 ^b^	31.9 ± 5.2	25.4 ± 3.2	18.5 ± 1.7
Proline	609.5 ± 211.5^a^	1603.0 ± 191.7^b^	2134.0 ± 248.5^c^	94.2 ± 16.6	104.6 ± 3.3	164.3 ± 21.9
Serine	601.8 ± 157.1^a^	1395.4 ± 79.4^b^	1914.5 ± 295.7^c^	139.8 ± 20.1	160.4 ± 7.4	220.8 ± 19.1
Threonine	93.6 ± 33.2^a^	136.3 ± 16.3 ^b^	96.3 ± 18.3 ^ac^	42.9 ± 7.7	51.4 ± 11.7	42.3 ± 6.1
Tryptophan	27.8 ± 6.0^a^	33.5 ± 4.2^b^	32.9 ± 4.8 ^ab^	8.4 ± 1.3	6.8 ± 0.7	4.9 ± 0.4
Tyrosine	82.9 ± 17.8^a^	102.8 ± 14.0^b^	101.7 ± 17.4^b^	26.3 ± 5.3	20.0 ± 1.7	13.8 ± 1.7
Valine	486.4 ± 146.6^a^	982.3 ± 125.7^b^	1642.5 ± 394.5^c^	79.8 ± 11.5	89.3 ± 4.9	124.9 ± 16.3

The concentrations of the amino acids arginine, ornithine, proline, serine, and valine measured in sperm from cold-stored milt samples, significantly increased with both storage time and temperature (*p* < 0.001). The concentrations of alanine, lysine, phenylalanine, and tyrosine were significantly increased in D4-2°C compared to D1-2°C (*p* < 0.05). However, there was no significant difference between D4-2°C and D4-8°C samples. The glutamate concentration was significantly higher in D4-8°C, compared to D1-2°C (*p* < 0.01). For the amino acids glutamine, histidine and tryptophan, the concentration was significantly higher in D4-2°C compared to D1-2°C (*p* < 0.05). However, there was no significant effect of increasing the storage temperature to eight degrees. Overall, there was a significant effect of cryopreservation for all amino acid concentrations except for asparagine in D4-8°C and ornithine in D1-2°C. For the cryopreserved samples, only the concentrations of alanine (*p* < 0.05) and GABA (*p* < 0.02) were significantly reduced during storage. No significant changes were observed after increasing the storage temperature to eight degrees. The results for metabolite concentrations in seminal plasma showed similar patterns to the sperm cell samples, where several metabolites were affected by storage conditions during cold storage (Supplementary Table S4). None of the metabolites in the seminal plasma of frozen-thawed samples were significantly affected by the storage conditions prior to cryopreservation.

### 3.8 Correlation of metabolites and fertilization rate

Correlations (Spearman correlation) between sperm metabolites and fertilization outcome using a sperm:egg ratio of 75 × 10^3^ is presented in Table 4. For the cold-stored samples, the highest positive correlations were found for asparagine (r_s_ = 0.88) and GABA (r_s_ = 0.86), while choline (r_s_ = −0.87) showed the highest negative correlation. For the cryopreserved samples, all the amines and several amino acids showed significant correlations to the fertilization outcome. The highest positive correlation was observed for GABA (r_s_ = 0.90), creatine (r_s_ = 0.88), and L-carnitine (r_s_ = 0.85). Arginine (r_s_ = −0.81) and choline (r_s_ = −0.78) showed the highest negative correlation.

**TABLE 4:** Spearman correlation coefficient values between metabolite concentrations in sperm cells and percentage of fertilized eggs (sperm:egg ratio of 75 × 10^3^) in Atlantic salmon (*Salmo Salar* L*.*) using cold-stored or the corresponding cryopreserved milt after cold storage. Only metabolites significantly (*p* < 0.05) different from zero are presented.

	Cold-stored	Cryopreserved
Amines		
Choline	−0.87	−0.78
Creatine	0.66	0.88
L-carnitine		0.85
Aminoacids		
Alanine	−0.71	0.76
Arginine	−0.76	−0.81
Asparagine	0.88	0.81
GABA	0.86	0.90
Glutamate	−0.67	
Glutamine		0.80
Glycine		0.83
Histidine		0.81
Isoleucine		0.69
Leucine		0.82
Lysine		0.78
Methionine		0.78
Ornithine		−0.64
Phenylalanine		0.79
Proline	−0.73	
Serine	−0.74	
Tryptophan		0.79
Tyrosine		0.72
Valine	−0.77	

### 3.9 Basic statistics of RRBS libraries

Overall, minimal variation was observed between libraries regarding retrieved clean data, mapping efficiency, and global CpG methylation levels (Table 5). Using Trim Galore default options, we were able to recover, on average, 99.7% of the reads, corresponding to 83.8% of the total bases in the original reads. Libraries mapped against the *Salmo salar* reference genome, showed on average 59.3% unique mapping efficiency. Furthermore, the libraries had a high degree of global methylation percentage (largely close to 87.3%). In this study, only CpGs with equal or greater than 10x coverage were considered for methylation analysis. The number of CpG_10X_ varied between samples from 2 million to 4.5 million Cs, corresponding to an average of 23.6% of all covered Cs in the libraries. To compare the methylation profiles, all samples were considered for correlation and cluster analyses based on CpG_10X_ methylation values. Pearson’s correlation revealed a high similarity between samples (Pearson’s correlation coefficient > 0.96) and cluster analysis showed that test and control groups from the same individuals always were clustered together (Supplementary Figure S1; Supplementary Table S5).

**TABLE 5:** An overview of basic statistics for RRBS libraries. Sample IDs reflecting Atlantic salmon (*Salmo salar* L.) individuals (A, B, C, D), days of milt storage (D1 and D4), and temperature of storage (2°C and 8°C), with subsequent cryopreservation (Cryo). Libraries, quality filtered and trimmed using trim-galore, and the number of reads and bp were compared with pre-trimmed data. Mapping efficiency shows the percentage of uniquely mapped clean reads with the reference genome. CpG methylation shows the percentage of global methylation in clean reads. The number of CpG_10X_ quantified in clean reads after trimming and the percentage of CpG_10X_ were calculated relative to all Cs with any coverage in libraries.

Sample ID	Recovered data after trimming (percentage of reads—bp)	Mapping efficiency	Global CpG methylation (%)	Number of CpG_10x_ (percentage of CpG_10x_)
A-D1-2°C	99.6–84.9	58.3	87.7	2550411 (23.8)
B-D1-2°C	99.8–84.6	58.0	87.0	2778942 (23.4)
C-D1-2°C	99.7–82.3	58.7	87.6	3067272 (26.3)
D-D1-2°C	99.7–83.7	57.5	87.2	2669483 (24.0)
A-D1-2°C/Cryo	99.8–85.0	60.4	87.5	2648800 (18.2)
B-D1-2°C/Cryo	99.8–84.8	60.7	86.9	4542774 (28.9)
C-D1-2°C/Cryo	99.8–85.2	61.8	87.6	4596704 (29.4)
D-D1-2°C/Cryo	99.8–84.9	60.7	87.2	2416751 (16.0)
A-D4-2°C	99.7–82.4	57.5	87.7	4202235 (32.8)
B-D4-2°C	99.7–82.9	58.0	87.3	3398408 (28.0)
C-D4-2°C	99.6–80.5	57.3	87.5	3478501 (30.2)
D-D4-2°C	99.8–83.5	58.0	87.2	3521833 (29.4)
A-D4-8°C	99.7–85.4	61.8	87.4	3389145 (21.5)
B-D4-8°C	99.6–84.5	60.1	87.1	2204071 (15.5)
C-D4-8°C	99.7–83.8	61.6	87.6	2113378 (15.2)
D-D4-8°C	99.7–82.9	59.6	87.4	2019531 (15.3)

### 3.10 Differential methylation analysis

Differential methylation analysis was performed using CpG_10X_ after applying a linear regression model and 10% methylation cut-off as well as a *q*-value < 0.05. This resulted in identifying diverse numbers of DMCs (from 226 to 3483), with different levels of methylation difference ranging from −64% to 59% among different experiments (Table 6). In experiments B and E, test groups were less methylated compared to control groups, while in experiments C and D an opposite trend was observed.

**TABLE 6:** An overview of the number of filtered DMCs and range of differences in methylation level identified in Atlantic salmon (*Salmo salar* L.) milt samples from different experiments (Exp A to E). Both hypo- and hyper-methylated Cs percentages refer to differences in test samples compared to control. For instance, in Exp A, 49 percent of mutual Cs in test individuals were less methylated compared to the same Cs in the control group.

	Exp A	Exp B	Exp C	Exp D	Exp E
Total number of filtered DMCs (range of methylation difference in percentage)	314 (−47 to 56)	2501 (−50 to 56)	1679 (−40 to 40)	3483 (−64 to 59)	226 (−40 to 41)
Percentage of hypomethylated Cs	49.0	69.0	41.8	34.7	59.2
Percentage of hypermethylated Cs	51.0	31.0	58.2	65.3	40.8

### 3.11 Annotation of DMCs with gene and CpG features

Both hypo- and hyper-methylated Cs in all experiments were separately annotated with different genomic and CpG features. Considering gene features, results showed that the majority of DMCs were annotated with intergenic regions followed by introns (Table 7). Furthermore, a higher percentage of DMCs (both in hypo- and hyper-methylated groups) was annotated to promoter regions compared to exons. Regarding CpG features, on average, at least 95% of filtered DMCs in both hypomethylation and hypermethylation groups were annotated within regions outside of CpG islands (CGI)/CpG shores.

**TABLE 7:** An overview of the distribution (in percentage) of filtered DMCs identified in different experiments (Exp A to E) across gene and CpG features in the Atlantic salmon (*Salmo salar* L.) genome. Numbers indicate the percentage of annotated DMCs.

		Gene features				CpG features		
		Intergenic regions	Promoter	Exon	Intron	CGI	Shore	Other
Exp A	Hypo	56	4	2	38	5	4	91
	Hyper	58	8	5	29	2	3	95
Exp B	Hypo	54	5	4	37	1	3	96
	Hyper	52	7	4	37	1	3	96
Exp C	Hypo	54	8	2	36	3	2	95
	Hyper	56	6	3	35	2	2	96
Exp D	Hypo	56	7	4	33	2	3	95
	Hyper	53	4	4	39	2	3	95
Exp E	Hypo	58	4	3	35	1	1	98
	Hyper	41	9	3	47	0	2	98

Hypo, hypomethylated Cs; Hyper, hypermethylated Cs; CGI, CpG island; Shore, CpG shore.

### 3.12 Functional annotation analysis

**FIGURE 2:**
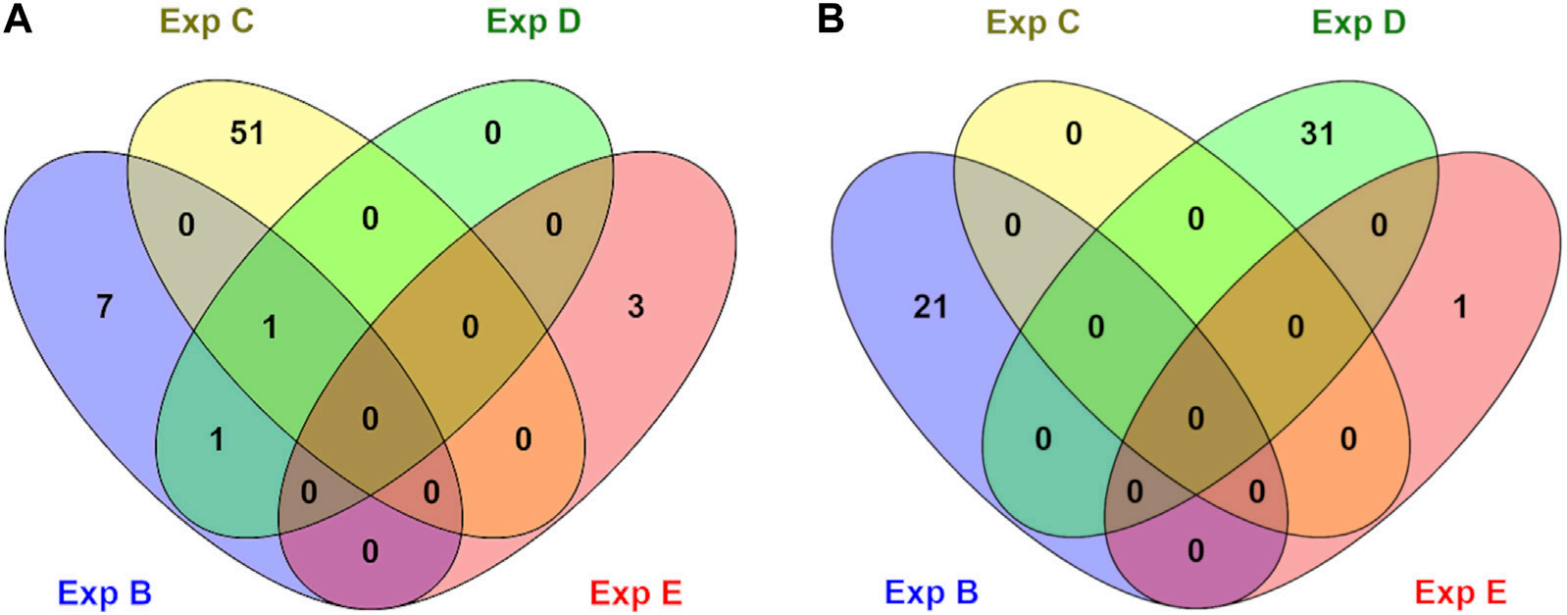
An overview of numbers of exclusive and shared identified significant GO terms in hypo **(A)** and hyper **(B)** methylated groups in different experiments (B, C, D, and E). No significant terms were identified for experiment A.

Genes whose TSSs were annotated with DMCs were first identified and subsequently subjected to functional annotation analysis. In the current study, significantly enriched terms were observed for all experiments, except experiment A. For each experiment, the top five significant terms and pathways per group (hypo and hyper) are shown in Table 8. A full list of significant terms and pathways can be found in (Supplementary Table S6). We observed the highest number of significant terms and pathways for experiment C, followed by D, B, and E (Figure 2). The analysis clearly showed that a wide variety of significant terms related to signaling, membrane structure, ion exchange, energy metabolism, cell adhesion, hormone, and embryo development can be linked to genes proximal to DMCs (Supplementary Table S6). The term entitled leucine-rich repeats, was the only mutually identified term in the hypo group of experiments B, C, and D (Figure 2A). No terms or pathways were found mutual among hyper groups between experiments (Figure 2B).

**TABLE 8:** An overview of functional annotation analysis for annotated genes associated with filtered DMCs in experiments B, C, D and E. No significant terms were enriched for experiment A. The *p*-value was benjamini corrected to correct for multiple testing.

		Database	Enriched term	Adjusted *p*-value
Exp B	Hypo	INTERPRO	IPR000233:Cadherin, cytoplasmic domain	1.38E-04
		INTERPRO	IPR027397:Catenin binding domain	0.0021
		INTERPRO	IPR003598:Immunoglobulin subtype 2	0.0144
		INTERPRO	IPR000863:Sulfotransferase domain	0.0151
		INTERPRO	IPR003591:Leucine-rich repeat, typical subtype	0.0151
	Hyper	INTERPRO	IPR000233:Cadherin, cytoplasmic domain	4.14E-05
		INTERPRO	IPR027397:Catenin binding domain	5.62E-05
		INTERPRO	IPR014868:Cadherin prodomain	0.0017
		GOTERM_BP	GO:0071346∼cellular response to interferon-ϒ	0.0027
		INTERPRO	IPR020894:Cadherin conserved site	0.0083
Exp C	Hypo	GOTERM_MF	GO:0004689∼phosphorylase kinase activity	3.11E-12
		KEGG_PATHWAY	sasa04626: Plant-pathogen interaction	3.07E-07
		KEGG_PATHWAY	sasa04016: MAPK signaling pathway - plant	5.17E-07
		KEGG_PATHWAY	sasa04070: Phosphatidylinositol signaling system	3.74E-05
		KEGG_PATHWAY	sasa05133: Pertussis	7.61E-05
Exp D	Hypo	INTERPRO	IPR003598:Immunoglobulin subtype 2	0.0488
		INTERPRO	IPR003591:Leucine-rich repeat, typical subtype	0.0488
	Hyper	GOTERM_MF	GO:0004689∼phosphorylase kinase activity	2.00E-08
		UniProt_BP	KW-0524∼Neurogenesis	1.91E-04
		KEGG_PATHWAY	sasa04016:MAPK signaling pathway - plant	2.27E-04
		KEGG_PATHWAY	sasa04626:Plant-pathogen interaction	3.94E-04
		KEGG_PATHWAY	sasa04745:Phototransduction - fly	0.0016
Exp E	Hypo	INTERPRO	IPR020479:Homeodomain, metazoa	0.0036
		INTERPRO	IPR017970:Homeobox, conserved site	0.0325
		GOTERM_MF	GO:0000981∼RNA polymerase II transcription factor activity, sequence-specific DNA binding	0.0448
	Hyper	UniProt_BP	KW-0119∼Carbohydrate metabolism	0.00340

BP; biological process, MF; molecular functions.

## 4 Discussion

The main aim of the present study was to assess if cold storage of milt from Atlantic salmon prior to cryopreservation compromises post-thaw sperm quality and fertilizing potential, and if metabolomics and epigenetic signatures may reveal further aspects of milt quality evaluation. The results show that the fertilizing capacity of milt is preserved when stored for up to 4 days at two degrees. As hypothesized, cold storage for 4 days at eight degrees was detrimental to sperm quality and reduced the fertilization capacity of milt. Further, the results show that cryopreservation should be conducted shortly after milt collection, as milt cryopreserved after cold storage for 4 days exhibited reduced fertilization upon thawing. The negative effect of storage time, temperature, and cryopreservation was more pronounced when the sperm:egg ratio was reduced from 500 × 10^3^ to 75 × 10^3^ sperm per egg. Most of the *in vitro* sperm quality parameters were negatively influenced by storage time, temperature, and subsequent freezing/thawing. These findings are in agreement with previous studies reporting that cold storage and cryopreservation of Atlantic salmon sperm may affect *in vitro* sperm quality and fertilizing ability (Díaz et al., 2019; Kommisrud et al., 2020; Erraud et al., 2022).

The sperm viability decreased significantly during cold storage for 4 days. This is in agreement with previous studies in fish (Trigo et al., 2015; Kommisrud et al., 2020), and also reflects the observed decline in fertilizing ability during cold storage for 4 days at eight degrees. Interestingly, Kommisrud et al. (2020) found a small positive effect of storage on fertilization even though the viability was reduced during 6 days of cold storage. The discrepancy with our results may be explained by the lower sperm:egg ratio used in our study. The frozen-thawed samples had significantly lower sperm viability compared to cold-stored samples. This is congruent with previous reports in mammals (Gürler et al., 2016; Narud et al., 2021a) and fish (Díaz et al., 2019; Lee-Estevez et al., 2019).

The presented CASA results on TM and PM are significantly lower for cold-stored milt than otherwise reported for Atlantic salmon (Dziewulska et al., 2010; Parodi et al., 2017). For frozen-thawed milt, our results for TM and PM are higher for day one than previously reported by others (Dziewulska et al., 2011), while comparable to others when cold-stored for 4 days prior to cryopreservation (Kommisrud et al., 2020). There are several factors that may affect motility results, including temperature and sperm activation procedures (Caldeira and Soler, 2018), as well as differences between CASA systems and the settings used for the analysis (Boryshpolets et al., 2013).

For both cold-stored and frozen-thawed samples, there was a significant decline in TM, PM, and ATP levels from day one to day four of storage. Surprisingly, the frozen-thawed sperm cells did not show significantly reduced motility and ATP levels compared to cold-stored samples, in disagreement with previous results reported by others (Dziewulska et al., 2011; Figueroa et al., 2016; Kommisrud et al., 2020). Interestingly, the velocity parameters and LIN and STR were significantly higher in samples cryopreserved after cold storage compared to those stored cold. This is in disagreement with previous studies, reporting that the kinematic parameters decrease after cryopreservation (Dziewulska et al., 2011; Figueroa et al., 2016; Kommisrud et al., 2020). Our results may have been influenced by the activation procedure, which was identical for fresh and cryopreserved samples. A dedicated activation procedure for the fresh milt could have been more optimal and given results more in compliance with other reports. However, the motility values for the different samples are comparable relative to each other within storage conditions. This is also reflected by the correlation analysis, where the post-thaw *in vitro* motility parameters displayed a greater correlation with fertilization rates than for the cold-stored samples.

Sperm DNA integrity is crucial for successful fertilization and embryo survival (Pérez-Cerezales et al., 2010; Kumaresan et al., 2020). Both cold-stored and frozen-thawed milt showed significantly higher percentages of DFI and HDS in samples stored for 4 days at two and eight degrees, compared to samples analyzed on day one. Furthermore, frozen-thawed sperm had significantly higher levels of fragmented DNA compared with cold-stored sperm. This is congruent with the literature, reporting that both cold-stored and cryopreserved milt are prone to sperm DNA damage (Cabrita et al., 2005; Pérez-Cerezales et al., 2011; Contreras et al., 2020).

All the *in vitro* post-thaw parameters assessed in this study were correlated with fertilization rates for 75 × 10^3^ sperm per egg. The highest correlations were found for the motility parameters TM and PM, ATP content, and the kinematic parameters VAP, VCL, and VSL, consistent with results obtained by others (Dziewulska et al., 2011; Figueroa et al., 2016; Kommisrud et al., 2020). Also, the DNA integrity parameters DFI and HDS were significantly negatively correlated with fertilization rates. As expected, the correlations were weaker with 500 × 10^3^ sperm per egg. Cryopreservation of cold-stored samples resulted in a significant reduction of fertilized eggs compared to cold-stored samples, except for day one using the sperm:egg ratio 500 × 10^3^. However, the relatively low fertilization rate obtained for day one with cold-stored milt might be explained by poor egg quality observed this day.

Previous studies have investigated the metabolome of fish milt and seminal plasma (Dietrich et al., 2019b; Tsentalovich et al., 2020). However, to our knowledge, this is the first study investigating sperm metabolites in relation to fertilizing potential of cold-stored and cryopreserved salmon milt. Overall, our results showed that the different storage conditions of cold-stored milt significantly affected the level of amines and several amino acids. In frozen-thawed samples, only a few metabolites were affected by storage conditions prior to cryopreservation. The process of freezing and thawing milt significantly reduced the levels of all amines and most of the amino acids studied. Our *in vitro* sperm quality analysis showed a significant reduction in sperm viability after freezing and thawing. Thus, there was a higher percentage of dead sperm cells in the cryopreserved samples analyzed for targeted metabolites compared to the cold-stored samples. It is possible that the damaged sperm membranes have leaked intracellular metabolites causing the significant reduction in metabolite concentrations observed after cryopreservation. However, the seminal plasma samples did not necessarily show a corresponding increase in metabolite concentration. This may be explained by the metabolite concentrations of seminal plasma samples of cold-stored and cryopreserved milt being not directly comparable. The cryopreserved samples were diluted based on a standardized cell number while the cold-stored samples were diluted 1:1 independent of the sperm number. Thus, due to a higher dilution factor for frozen-thawed samples, the concentrations of metabolites in the seminal plasma samples will be lower. For future studies, the dilution of cold-stored and cryopreserved samples should be standardized such that the metabolite concentrations of cold-stored and frozen-thawed milt samples can be compared directly.

Metabolites such as different amino acids and amines have been shown to act as antioxidants, protecting sperm cells from oxidative stress during freezing and thawing (Davoodian et al., 2017; Dietrich et al., 2019b; Ugur et al., 2019). However, the exact mechanisms of metabolites as cryo-protectants have not been clearly understood and there are few studies documenting the role of metabolites during freezing and thawing of milt. Interestingly, after cryopreservation, the level of L-carnitine was significantly reduced in the samples stored for 4 days compared to day one samples. Furthermore, the level of L-carnitine was positively correlated with fertilization rate of cryopreserved samples. In mammals, L-carnitine has been shown to be highly concentrated in the epididymis and sperm (Jeulin and Lewin, 1996). It is essential for energy metabolism and is involved in sperm motility, sperm maturation and fertilization. Furthermore, L-carnitine is shown to protect sperm cells against oxidative damage (Abdelrazik and Agarwal, 2009; Aliabadi et al., 2012). Thus, one hypothesis for this reduction in L-carnitine is that more L-carnitine has been utilized to protect the sperm cells from reactive oxygen species during the 4 days of cold-storage. For future studies, it would be interesting to further investigate the effect of L-carnitine supplementation on milt quality during storage and cryopreservation. Overall, our results show that storage conditions and cryopreservation of salmon milt affect the concentration of sperm metabolites. Furthermore, several of the metabolites studied were highly correlated with the fertilization ability of salmon milt. Metabolomics of sperm and seminal plasma has thus been shown applicable for Atlantic salmon milt, and a possible tool for broader fertility research. Further studies applying a larger number of individuals are needed to increase knowledge of the fish sperm metabolome, its relationship with fertilizing ability, and the possible role of metabolites as cryo-protectors.

In the current study, the average global sperm DNA methylation level after different milt handling regimes was 87.3%. Similar global hypermethylated status was previously reported for Atlantic salmon milt (Wellband et al., 2021), fertilized salmon eggs (Moghadam et al., 2017) and Rainbow trout milt (Gavery et al., 2018). We previously reported 33%–43% of global methylation levels in boar (Khezri et al., 2019) and bull sperm cells (Khezri et al., 2020; Narud et al., 2021b) using the RRBS technique. This indicates different parental DNA methylation patterns among mammalian species and salmonids. Although, in this study, the methylation profiles of CpG_10x_ were similar between samples, clustering the control and test samples from the same individuals together might also be explained by the low number of samples.

In this study the overall distribution trend of filtered DMCs over gene and CpG features was similar to the trend previously seen in boar (Khezri et al., 2019) and bull (Khezri et al., 2020). Similar distribution and annotation rates previously reported for Atlantic salmon (Moghadam et al., 2017; Robinson et al., 2019). However, the percentages of annotated filtered DMCs with intron in salmon were considerably higher compared to bull and boar reported previously by our group and might be explained by the interspecies differences. Worth mentioning that the differences in percentages of annotated filtered DMCs with introns and CGIs among salmon and bull were smaller than the differences in salmon and boar for the same regions. Several factors could explain this observation. Firstly, the genome size of salmon (2.8 Gb) and bull (2.7 Gb) is bigger than pig (2.5 Gb) and secondly, we sequenced the libraries for both salmon and bull in paired-end mode vs*.* single-end mode in boar. This potentially provides more coverage for the analyses.

To our knowledge, this is the first study investigating the effects of storage on sperm DNA methylation patterns in Atlantic salmon. A previous study documented the sperm DNA methylation pattern in European carp (*Cyprinus carpio*) and revealed that similar to our study (Exp A), refrigeration of milt for 24 h significantly increased methylation levels (Cheng et al., 2021). This is similar to the results presented here for Exp A, although we observed a slight hypermethylation. It seems reasonable to consider milt handling and storage time as environmental stress factors. Previous studies have shown that DNA methylation in salmonid sperm cells is highly dynamic and can be influenced by different environmental factors. For instance, sperm cells taken from wild salmon showed very distinct epigenetic signatures compared to sperm cells from hatcher-reared Atlantic salmons (Rodriguez Barreto et al., 2019) and rainbow trout (Nilsson et al., 2021).

Results in this study revealed no significant enriched pathways and Go terms for Exp A. This is comparable with similar and not significant fertilization rates between D1–2°C and D1–2°C/Cryo for both sperm:egg ratios. Therefore, both experiments indicate that cryopreservation within 1 day of milt collection, might be the optimal protocol among tested methods. It is worth mentioning that optimization of the extender formula used for cryopreservation, might have some effects on sperm DNA methylation levels. For instance, it has been shown that cryoprotectant agents decreased the global sperm DNA methylation level in Tambaqui fish, *Colossoma macropomum* (de Mello et al., 2017).

Results in this study documented that milt handling and storage could significantly change site-specific methylation levels proximal to genes involved in important biological pathways. We observed a significant enrichment of terms related to hormones (renin, oxytocin, insulin, GnRH, aldosterone, estrogen) and signaling, in both hypo group of exp C and hyper group of exp D. Terms reflecting developmental pathways such as melanogenesis, oocyte meiosis (hypo group of exp C), neurogenesis, angiogenesis and nervous system development (hyper group of exp D) were also identified. This is in agreement with previously published results where rearing condition changed sperm DNA methylation status close to genes involved in neuronal development in Atlantic salmons (Rodriguez Barreto et al., 2019). Similar to our study, previously published results in rainbow trout showed that important biological pathways such as development and signaling can be linked with differentially methylated regions in sperm cells taken from wild and hatchery-reared rainbow trout (Nilsson et al., 2021).

Furthermore, we observed a significant overrepresentation of GO terms related to immune response (hypo groups of both exp B and D) and calcium signaling in hypo group of exp C and hyper group of exp D. This is in line with previously published results where authors showed epigenetic modifications induced by hatchery rearing condition in pacific salmon (Le Luyer et al., 2017). Furthermore, our analysis showed that the GO term homeobox protein is significantly enriched in hypo group of Exp E. Different protein members of the Homeobox family also were significantly enriched following changes in sperm DNA methylation in wild and hatchery-reared rainbow trout (Gavery et al., 2018). It has been shown that a homeobox protein expressed in the male reproductive system is involved in developmental events in different species including salmon and cattle (Cánovas et al., 2014; Mobley et al., 2021).

GO terms representing the membrane integrity and cell adhesion process were specifically enriched in Exp B. Leucine-rich repeats (LPR) was the only term significantly enriched in the hypo groups of experiments B, C, and D. The LPR motifs have been reported in a variety of proteins involved in different biological processes such as signaling, cell adhesion, apoptosis, and the immune response (Ng and Xavier, 2011). Interestingly, in this study, leucine was identified as an amino acid whose concentration in sperm cells changed dramatically upon cryopreservation.

The research to date has tended to focus on environmental stressors such as rearing condition effects on epigenetic modification, rather than milt handling effects. The current results highlight the importance of handling and storage of Atlantic salmon milt and their potential to induce epigenetic modifications in sperm cells. The current study showed that milt handling and storage changed DNA methylation patterns close to genes involved mostly in developmental processes. Hence, it could conceivably be hypothesized that developmental pathways are at greater risk following incorrect milt handling and storage. The same conclusion can be drawn based on previous publications where different rearing conditions were tested. Therefore, one could speculate that environmental factors have the potential to alter DNA methylation in Salmonid sperm cells (whether the semen sample is exposed to those factors or the donor) in regions proximal to genes that are potentially involved in the development. More broadly research is needed to determine these effects in the offspring of Atlantic salmon.

In this study, we aimed to create contrasts to explore the potential value of metabolomics and epigenetics on Atlantic salmon sperm and milt. It was successfully shown that storage duration and corresponding cryopreservation influenced metabolites and sperm DNA methylation signatures. Further studies should include shorter preservation intervals to reveal the possibility of applying an extended period of storage before cryopreservation.

In conclusion, the fertilization capacity of cold-stored milt from Atlantic salmon is preserved when stored for up to 4 days at two degrees. Cold storage for 4 days at eight degrees and corresponding cryopreservation was detrimental to sperm quality and influenced levels of milt metabolites and DNA methylation signatures. Cryopreservation of milt stored for 1 day at two degrees do not compromise either fertilization ability or sperm DNA methylation signatures.

## Data Availability

The datasets presented in this study can be found in online repositories. The names of the repository/repositories and accession number(s) can be found below: https://www.ebi.ac.uk/ena, PRJEB61229.
