# Supplementary material for: Sperm quality parameters, fertilizing potential, metabolites, and DNA methylation in cold-stored and cryopreserved milt from Atlantic salmon (Salmo salar L.)
Source: Front Genet. 2023 Aug 24;14:1199681. doi: 10.3389/fgene.2023.1199681 (PMC10483119; doi:10.3389/fgene.2023.1199681)
Supplement: Supplementary file 3 [file Table1.DOCX]

Supplementary Material

**Sperm quality parameters, fertilizing potential, metabolites, and DNA methylation in cold-stored and cryopreserved milt from Atlantic Salmon (*Salmo salar* L.)**

**Birgitte Narud^1*^, Abdolrahman Khezri^1^, Teklu T. Zeremichael^1^, Anne-Lene Eriksen^2^, Inger S. Grevle^2^,** **Anna Nordborg^3^, Geir Klinkenberg^3^, Robert C. Wilson^1^, Elisabeth Kommisrud^1^**

*** Correspondence:** Birgitte Narud: [birgitte.narud@inn.no](mailto:birgitte.narud@inn.no)

# Supplementary Figures and Tables

**Table S1.** Method parameter (multiple-reaction monitoring) and instrument settings for LC-MS/MS determination of amino acids.

|  | Precursor | Product ion | CV (V) | RT (min) | Internal standard |
| --- | --- | --- | --- | --- | --- |
| Alanine | 260 | 171/116 | 32/65 | 5.7 | Alanine-D3 |
| Arginine | 345.2 | 175/171 | 20/32 | 2.9 | Arginine-13C6 |
| Asparagine | 303.2 | 171/116 | 20/60 | 3.1 | - |
| Glutamate | 318.1 | 171/116 | 36/65 | 5.2 | Glutamate-D5 |
| Glycine | 245.9 | 171/116 | 30/54 | 4.7 | Glycine-13C2 |
| Glutamine | 317.2 | 171/116 | 26/62 | 4.1 | Glutamine-13C5 |
| GABA | 274.2 | 171/116 | 28/60 | 5.7 | GABA-D6 |
| Histidine | 326.6 | 171/116 | 26/68 | 1.8 | Histidine-13C6 |
| Isoleucine | 302.2 | 171/116 | 20/64 | 9.9 | - |
| Leucine | 302.1 | 171/116 | 28/70 | 10.2 | Leucine-D10 |
| Lysine | 487.2 | 171/116 | 32/80 | 6.5 | Lysine-D10 |
| Methionine | 320 | 171/116 | 26/68 | 7.7 | Methionine-13CD3 |
| Ornithine | 173.1 | 171/116 | 44/80 | 6.1 | Ornithine-D6 |
| Phenylalanine | 336.1 | 171/116 | 32/72 | 11.4 | Phenylalanine-D5 |
| Proline | 286.1 | 171/116 | 24/70 | 6.0 | Proline-D3 |
| Serine | 276.1 | 171/116 | 24/58 | 4.2 | Serine-D3 |
| Threonine | 290.1 | 171/116 | 32/65 | 5.3 | - |
| Tryptophan | 375.1 | 171/116 | 32/76 | 13.1 | Trypthophan-D5 |
| Tyrosine | 352.2 | 171/116 | 20/70 | 7.6 | Tyrosine-13C9 |
| Valine | 288 | 171/116 | 16/66 | 7.8 | Valine-D8 |

Instrument settings; Capillary voltage (CV) 3 kV, Nozzle voltage 500 V, Nebulizer pressure: 40 psi, drying gas flow: 14 L/min, gas temperature: 160 °C, Fragmentor voltage: 380 V, Cell acceleration voltage 3 V, Sheath gas temperature: 400 °C, sheath gas flow: 11 L/min, iFunnel positive high/low pressure RF: 190/100, and negative high/low pressure RF: 150/60. GABA = ɣ-Aminobutyric acid

**Table S2.** Method parameter (multiple-reaction monitoring) and instrument settings for LC-MS/MS determination of amines.

|  | Precursor | Product ion | CV (V) | Cell acceleration voltage (V) | Internal standard |
| --- | --- | --- | --- | --- | --- |
| Choline | 104.1 | 60/45 | 18/25 | 3 | D13-choline |
| L-Carnitine | 162.2 | 103/60 | 15/17 | 3 | D3-carnitine |
| Creatine | 132.1 | 90/44 | 10/25 | 3 | - |

Instrument settings; Capillary voltage (CV) 2 kV, Nebulizer pressure: 30 psi, drying gas flow: 14 L/min, gas temperature: 250 °C, Fragmentor voltage: 380 V, Sheath gas temperature: 400 °C, sheath gas flow: 10 L/min, iFunnel positive high/low pressure RF: 120/60, and negative high/low pressure RF: 90/60.

**Table S3.** Correlation coefficient (corr) and p-values for the correlations between sperm parameters and fertilization outcome using sperm:egg ratio of 75x10^3^ and 500x10^3^ assessed for Atlantic salmon males (n = 8). Only significant correlations (p<0.05) are presented.

|  | Cold-stored | | Cryopreserved | | | |
| --- | --- | --- | --- | --- | --- | --- |
|  | 75x10^3^ | | 75x10^3^ | | 500x10^3^ | |
| *In vitro* parameters | Corr | p-value | Corr | p-value | Corr | p-value |
| TM | 0.65 | <.001 | 0.96 | <.0001 | 0.83 | <.0001 |
| PM | 0.66 | <.001 | 0.96 | <.0001 | 0.83 | <.0001 |
| VAP | 0.67 | <.001 | 0.91 | <.0001 | 0.85 | <.0001 |
| VCL | 0.68 | <.001 | 0.91 | <.0001 | 0.84 | <.0001 |
| VSL | 0.63 | <.01 | 0.91 | <.0001 | 0.84 | <.0001 |
| STR | 0.43 | <.05 | 0.85 | <.0001 | 0.76 | <.0001 |
| LIN | 0.53 | <.01 | 0.89 | <.0001 | 0.83 | <.0001 |
| Viable | 0.58 | <.01 | 0.89 | <.0001 | 0.79 | <.001 |
| DFI | -0.66 | <.001 | -0.82 | <.0001 | -0.60 | <.002 |
| HDS | -0.63 | <.01 | -0.66 | <.001 | -0.51 | <.05 |
| ATP | 0.67 | <.001 | 0.93 | <.0001 | 0.82 | <.0001 |

TM = total motility, PM = progressive motility, VAP = velocity average path, VCL = velocity curvilinear, VSL = velocity straight-line, STR = straightness, LIN = linearity, DFI = DNA fragmentation index, HDS = High DNA stainable

**Table S4.** Mean and standard deviation for amine and amino acid concentrations (µM) in seminal plasma of cold-stored and the corresponding cryopreserved milt samples collected from Atlantic salmon (n=4). Different superscripts denote significant differences (p<0.05) between storage days and temperature within cold-stored and cryopreserved samples based on a linear mixed model.

|  | Supernatant/seminal plasma | | |
| --- | --- | --- | --- |
|  | Day 1 | Day 4, 2 °C | Day 4, 8 °C |
| *Cold-stored* |  |  |  |
| Choline | 89.8 ± 10.6 ^a^ | 133.3 ± 20.6 ^b^ | 157.0 ± 16.1 ^c^ |
| Creatine | 1021.8 ± 344.5 ^a^ | 1674.8 ± 424.2 ^b^ | 3120.3 ± 502.8 ^c^ |
| L_Carnitine | 30.3 ± 5.6 ^a^ | 45.5 ± 7.9 ^b^ | 77.5 ± 16.9 ^c^ |
|  |  |  |  |
| Alanine | 75.6 ± 18.6 ^a^ | 182.0 ± 55.4 ^b^ | 524.5 ± 144.2 ^c^ |
| Arginine | 462.5 ± 71.6 ^a^ | 2360.5 ± 658.3 ^b^ | 10074.4 ± 3126 ^c^ |
| Asparagine | 1.3 ± 0.6 ^a^ | 1.1 ± 0.5 ^ab^ | 0.8 ± 0.4 ^b^ |
| GABA | 3.4 ± 0.7 ^a^ | 5.0 ± 0.8 ^b^ | 8.7 ± 1.8 ^c^ |
| Glutamate | 285.1 ± 51.9 ^a^ | 383.0 ± 63.2 ^b^ | 518.3 ± 86.7 ^c^ |
| Glutamine | 71.7 ± 25.0 ^a^ | 149.1 ± 48.7 ^b^ | 263.6 ± 41.8 ^c^ |
| Glycine | 583.3 ± 300.0 ^a^ | 1424.5 ± 436.5 ^b^ | 3178.9 ± 469.6 ^c^ |
| Histidine | 35.6 ± 8.5 ^a^ | 64.2 ± 17.7 ^b^ | 102.8 ± 26.3 ^c^ |
| Isoleucine | 42.9 ± 10.8 ^a^ | 72.8 ± 28.3 ^b^ | 92.7 ± 17.1 ^c^ |
| Leucine | 108.0 ± 26.3 ^a^ | 202.2 ± 70.1 ^b^ | 303.3 ± 85.5 ^c^ |
| Lysine | 94.9 ± 19.1 ^a^ | 132.9 ± 38.1 ^ab^ | 227.5 ± 72.5 ^c^ |
| Methionine | 33.7 ± 9.1 ^a^ | 73.6 ± 21.1 ^b^ | 102.0 ± 25.3 ^c^ |
| Ornithine | 27.2 ± 6.6 ^a^ | 112.8 ± 36.9 ^b^ | 285.0 ± 79.2 ^c^ |
| Phenylalanine | 39.9 ± 9.2 ^a^ | 84.1 ± 21.3 ^b^ | 115.6 ± 26.8 ^c^ |
| Proline | 52.9 ± 17.0 ^a^ | 401.3 ± 123.9 ^b^ | 1641.0 ± 535.5 ^c^ |
| Serine | 172.9 ± 36.6 ^a^ | 587.4 ± 132.4 ^b^ | 1752.5 ± 480.8 ^c^ |
| Threonine | 107.9 ± 22.6 ^a^ | 162.0 ± 45.1 ^b^ | 181.9 ± 33.5 ^bc^ |
| Tryptophan | 8.4 ± 1.3 ^a^ | 16.7 ± 3.7 ^b^ | 22.4 ± 4.6 ^c^ |
| Tyrosine | 25.6 ± 7.0 ^a^ | 58.2 ± 16.3 ^b^ | 79.5 ± 17.8 ^c^ |
| Valine | 153.0 ± 27.5 ^a^ | 683.3 ± 154.2 ^b^ | 1476.0 ± 375.7 ^c^ |
| *Cryopreserved* |  |  |  |
| Choline | 10.7 ± 1.6 | 16.2 ± 1.6 | 18.1 ± 1.8 |
| Creatine | 419.6 ± 60.7 | 593.9 ± 64.8 | 602.2 ± 40.4 |
| L_Carnitine | 7.2 ± 1.3 | 11.8 ± 1.38 | 12.7 ± 1.5 |
|  |  |  |  |
| Alanine | 24.1 ± 3.8 | 51.5 ± 3.6 | 72.2 ± 6.5 |
| Arginine | 40.3 ± 6.3 | 588.2 ± 99.2 | 1329.1 ± 222.0 |
| Asparagine | N.A | N.A | N.A |
| GABA | 0.9 ± 0.03 | 1.3 ± 0.1 | 1.2 ± 0.1 |
| Glutamate | 46.7 ± 7.8 | 70.0 ± 2.6 | 81.3 ± 2.7 |
| Glutamine | 5.9 ± 1.8 | 12.5 ± 3.0 | 18.2 ± 3.0 |
| Glycine | 220.2 ± 69.9 | 357.9 ± 68.0 | 431.1 ± 36.7 |
| Histidine | 4.5 ± 0.6 | 8.8 ± 1.5 | 11.8 ± 2.5 |
| Isoleucine | 6.0 ± 1.4 | 8.4 ± 4.0 | 13.1 ± 7.3 |
| Leucine | 10.8 ± 1.8 | 17.1 ± 5.7 | 27.1 ± 8.1 |
| Lysine | 10.2 ± 1.3 | 19.5 ± 3.2 | 25.6 ± 5.9 |
| Methionine | 3.5 ± 0.8 | 8.5 ± 1.6 | 11.3 ± 2.5 |
| Ornithine | 3.4 ± 1.0 | 23.2 ± 7.5 | 48.1 ± 19.2 |
| Phenylalanine | 4.0 ± 0.5 | 9.1 ± 1.1 | 12.1 ± 2.1 |
| Proline | 7.2 ± 2.0 | 73.1 ± 12.7 | 160.7 ± 22.4 |
| Serine | 27.6 ± 2.9 | 116.6 ± 6.5 | 224.2 ± 26.3 |
| Threonine | 16.5 ± 3.3 | 32.0 ± 9.0 | 35.7 ± 13.2 |
| Tryptophan | 1.1 ± 0.1 | 2.2 ± 0.2 | 2.9 ± 0.5 |
| Tyrosine | 3.2 ± 0.5 | 7.3 ± 1.6 | 9.7 ± 2.0 |
| Valine | 13.8 ± 1.9 | 57.1 ± 10.4 | 122.8 ± 17.9 |

**Table S5**. Correlation analysis based on CpG10 methylation level in Salmo salar sperm samples treated differently in different experiments (A to E). Numbers in each cell represent the pairwise Pearson’s correlation scores. For detailed explanation of experiment conditions and test/ctrl samples please see table 1 in the main text.

**Table S6**: Full list of Go terms and associated KEGG pathways to genes whose TSS site was annotated with differentially methylated Cs (both hypo and hyper) in different experiments (A to E).

**Figure S1**. Hierarchical clustering analysis of samples based on CpG10 methylation level in *Salmo salar* sperm samples treated differently in different experiments (A to E). For detail explanation of experiment conditions and test/ctrl samples please see table 1 in the main text.

(The Supplementary Table S5, Table S6 and Figure S1 are uploaded as Additional files)
