# Supplementary figures and images for: Sperm quality parameters, fertilizing potential, metabolites, and DNA methylation in cold-stored and cryopreserved milt from Atlantic salmon (Salmo salar L.)
Source: Front Genet. 2023 Aug 24;14:1199681. doi: 10.3389/fgene.2023.1199681 (PMC10483119; doi:10.3389/fgene.2023.1199681)

## Slide 1
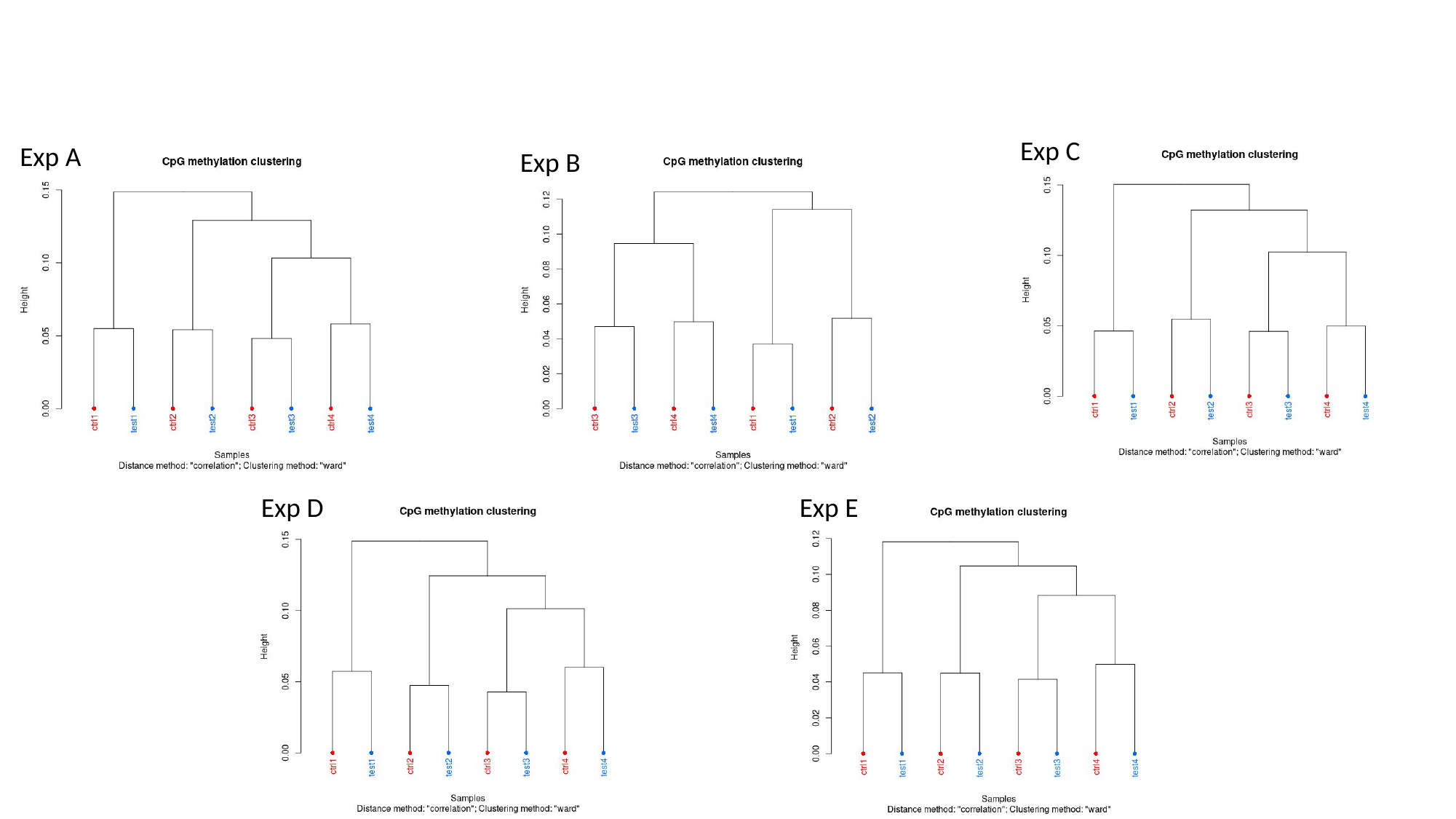

Exp C
Exp A
Exp B
Exp D
Exp E

Supplement: Supplementary file 4 [file Presentation1.PPTX]
